# Impact of maternally derived immunity on immune responses elicited by piglet early vaccination against the most common pathogens involved in porcine respiratory disease complex

**DOI:** 10.1186/s40813-022-00252-3

**Published:** 2022-03-16

**Authors:** Núria Martínez-Boixaderas, Laura Garza-Moreno, Marina Sibila, Joaquim Segalés

**Affiliations:** 1 IRTA. Programa de Sanitat Animal. Centre de Recerca en Sanitat Animal (CReSA), Campus de la Universitat Autònoma de Barcelona (UAB), 08193 Bellaterra (Barcelona), Catalonia Spain; 2 Ceva Salud Animal, Avenida Diagonal, 609-615, 9º Planta, 08028 Barcelona, Spain; 3 OIE Collaborating Centre for the Research and Control of Emerging and Re-Emerging Swine Diseases in Europe (IRTA-CReSA), 08193 Bellaterra, Catalonia Spain; 4 Departament de Sanitat i Anatomia Animals, Facultat de Veterinària, Universitat Autònoma de Barcelona, Bellaterra, 08193 Barcelona, Catalonia Spain; 5 Unitat mixta d’Investigació IRTA-UAB en Sanitat Animal, Centre de Recerca en Sanitat Animal (CReSA), Campus de la Universitat Autònoma de Barcelona (UAB), 08193 Bellaterra, Catalonia Spain

**Keywords:** Porcine respiratory disease complex (PRDC), Maternally derived immunity (MDI), Maternally derived antibodies (MDA), Vaccination, Interference, Piglet

## Abstract

**Background:**

Newborn piglets can trigger an elementary immune response, but the acquirement of specific antibodies and/or cellular immunity against pathogens before they get infected post-natally is paramount to preserve their health. This is especially important for the pathogens involved in porcine respiratory disease complex (PRDC) as they are widespread, fairly resistant at environment, and genetically variable; moreover, some of them can cause intrauterine/early life infections.

**Main body:**

Piglet protection can be achieved by either passive transfer of maternal derived immunity (MDI) and/or actively through vaccination. However, vaccinating piglets in the presence of remaining MDI might interfere with vaccine efficacy. Hence, the purpose of this work is to critically review the putative interference that MDI may exert on vaccine efficacy against PRDC pathogens. This knowledge is crucial to design a proper vaccination schedule.

**Conclusion:**

MDI transferred from sows to offspring could potentially interfere with the development of an active humoral immune response. However, no conclusive interference has been shown regarding performance parameters based on the existing published literature.

## Background

Suidae species are characterized by a six-layered epitheliochorial placenta [1] that, unless it is damaged during gestation [2] prevents leaking of large molecules like immunoglobulins from the sow to the foetuses [3]. However, foetuses can produce their own antibodies against antigens in the last third of gestation [4–6]. These antibodies are considered as part of the innate immune system [7] and are considered as “natural antibody repertoire”. Although they might play a protective role for the newborn pig [6], their response is weak [8] and little is known about their specificity and affinity [9]. On the other hand, piglets are born with functional immune cells and extracellular components able to respond to infections [10, 11]. However, due to limited or no external antigenic stimuli during foetal life, these components are usually immature at birth. Therefore, neonates are not fully immunologically competent.

Since the complete adaptative protective immune response of the piglet needs around four weeks to be established [12, 13], the protection of the newborn piglet against infectious agents is dependent on the acquirement of maternally derived immunity (MDI) from colostrum and milk [14].

The amount of MDI transferred from sows to their offspring is determined by the sow’s immunity level at the time of parturition, the timing of colostrum intake and the volume of colostrum ingested [15]. Strengthening sow herd immunity against specific diseases through exposure and/or vaccination is a useful management tool for ameliorating clinical effects in piglets and delaying infection until the piglet immune system is fully prepared to respond [13].

The duration of MDI is rather variable among pathogens. Under field conditions, it is usually measured considering only one arm of the immune system, the humoral response. In this regard, two terms are used in the literature to refer to the persistence of such MDI: the duration of maternally derived antibodies (MDA) detected by means of serological tests and the rate of MDA decay. The duration of MDA (Table 1) refers to the age of the piglet at which their MDA levels fall below the limit of detection of the test [16], whereas the rate of decay, also called “half-life”, indicates the time required for a 50% decrease in MDA levels [17]. This latter measure is a constant value and would be the most appropriate one to compare data among studies. However, the MDA decay is reported in few studies and significant variation is provided depending on the study; for example, MDA half-life for *Actinobacillus pleuropneumoniae* was calculated as 11–15 days by Vigre et al. [18] and 28–42 days by Cruijsen et al. [19]. Therefore, the duration of MDA is the most widely used parameter among published studies. Nevertheless, it is important to consider that the duration of MDA is dependent on the initial concentration of MDA and the threshold of the serologic test used [20].

**Table 1:** Duration of maternally derived antibodies (MDA) for each infectious agent included in this review

Pathogen	V/NV sows^a^	Study facilities	Duration of MDA (in weeks)	References
SIV	NV	Experimental	7–8	[65]
	V	Experimental	10	[23]
	V	Experimental	9–14	[67]
	V	Field	7–10	[69]
	V	Experimental	10	[66]
	V	Experimental	13–16	[68]
PRRSV	V	Field	4	[79]
	NV	Experimental	4–8	[77]
	U	Field	6–10	[80]
	U	Field	2–8	[81]
	U	Field	6–8	[72]
	V	Experimental	7–11	[71]
	V	Experimental	7–8	[78]
	V	Experimental	3–8	[82]
PCV-2	NV	Experimental	4–11	[93]
	U	Experimental	4–11	[96]
	NV	Field	10	[95]
	V	Field	4–12	[60]
	V	Field	7–12	[21]
	NV	Experimental	8	[94]
*M. hyopneumoniae*	NV	Field	4–9	[20]
	V	Experimental	2–7	[109]
*A. pleuropneumoniae*	U	Experimental	9–12	[19]
	NV	Field	8–10	[122]
	NV	Experimental	2–8	[18]
	NV	Experimental	10	[123]
	NV	Field	3–7	[124]

^a^*V,* Vaccinated prior to farrowing; *NV,* No vaccinated prior to farrowing; *U,* Unknown

If the level of MDI wane before piglets’ immune system has reacted against a given pathogen, there is a non-protected timeframe or time-window where the piglet is highly susceptible to infection. Therefore, the desirable scenario is to vaccinate piglets prior to natural infection although it implies that vaccination is performed in presence of MDI for most pathogens. Depending on the levels of such MDI, a potential interference of vaccine uptake may happen, jeopardizing vaccine seroconversion and efficacy [21–25].

The objective of this review was to compile information on how MDI can affect vaccine efficacy against the most common swine pathogens involved in porcine respiratory disease complex (PRDC), namely Swine Influenza viruses (SIV), Porcine reproductive and respiratory syndrome virus (PRRSV), Porcine circovirus 2 (PCV-2), *Mycoplasma hyopneumoniae (M. hyopneumoniae)* and *Actinobacillus pleuropneumoniae (A. pleuropneumoniae)*.

## Maternally derived immunity (MDI)

### Components of MDI

Different antibody isotypes are present to varying proportions within colostrum and milk [26]. Immunoglobulins G (IgG) and M (IgM) are the most common isotypes in colostrum, whereas immunoglobulin A (IgA) is the most common one in milk [27]. Colostrum and milk also contain large numbers of cells that may vary depending on the mammary gland’s developmental stage and the sow’s physiologic and immunologic status [28]. Many of them are leukocytes, such as neutrophils, other granulocytes, and mostly antigen-experienced lymphoid cells [29–32] that participate in the cell-mediated immune (CMI) response. In addition, there are also other components that are thought to play an immunomodulatory role, such as the antibacterial protein lactoferrin, lysozyme and cytokines [33, 34].

### MDI transfer

The newborn systemic and mucosal immune systems are immature, with limited peripheral lymphoid cells, underdeveloped lymph nodes, rudimentary jejunal Peyer’s patches and a low number of effector and memory T-lymphocytes [11, 35, 36]. As indicated above, the newborn piglet does not receive neither produce antibodies against a specific pathogen during gestation, unless a potential intrauterine infection or damage of placentation occurs during the immunocompetence period (from around 70–80 days of gestation onwards) [37]. In such eventuality, the animal would be delivered already with antibodies against the specific pathogen. Noteworthy, one report suggested PCV-2 antibody placental barrier leakage from sow to fetus, mainly in those cases from which the sows had very high levels of antibody titers [2]. These authors hypothesized that antibody crossing might be associated with small damage to the placental barrier during the gestational period. If this happens only with PCV-2 antibodies or with any type of antibody is presently unknown.

Antibodies and immune cells are transferred from sow to piglet by the ingestion of colostrum and milk, and these can cross the intestinal barrier and reach the peripheral blood for a limited period. This way they can reach lymphatic and non-lymphatic tissues including mesenteric lymph nodes, and a variety of other tissues such as the spleen, liver, lungs and the duodenum and jejunum’s lamina propria and submucosal spaces [33, 38, 39]. Nevertheless, this absorption process differs slightly between antibodies and cells [13, 40].

The peak of antibody transfer occurs immediately after birth. Therefore, it is critical to ensure piglet suckling for at least the first 6 h of life [40]. Acquired antibodies remain intact in the neonatal digestive tract due to low proteolytic activity, which is further reduced by sow colostrum trypsin inhibitor [41]. Antibodies (IgG but not IgA or IgM) can pass through the intestinal mucosa even if the maternal source or donor species is different from the own mother (for example, newborn pigs have been proven to absorb cow antibodies [15]). This absorption can be done by two transcytosis mechanisms to penetrate the intestinal barrier: non-specific endocytosis or antibody specific neonatal Fc-receptors [42–45]. The ability to absorb MDA by the proximal to the distal part of the small intestine lasts only for a short period of time. Specifically, Murata & Namioka [46] concluded that duodenum uptake stops 2 h after birth, while the jejunum’s and ileum uptake stop 48 and 72 h after birth, respectively. This post-natal loss of absorption capability is known as “gut closure”. Finally, within 9 days of birth, the mucosal epithelium is completely replaced by intestinal epithelial cells incapable of transcytosis [47, 48]. Colostral IgG are detectable in the neonatal circulatory system from 48 h after birth onwards [49, 50].

In contrast, transference of colostral cells needs a minimum period of suckling from 12 to 20 h, and the absorption is accomplished through the intercellular space between the epithelial cells of intestinal mucosa [38]. Years ago it was considered that only maternally-derived cells from the biological mother could pass the intestinal mucosa of piglets [38, 39]. This assumption was supported by Bandrick et al. [40] who reported no evidence of immune cell reactivity in cross-fostered animals just after birth. Therefore, animals fostered by a substitute dam could be deficient in CMI [40]. In contrast, new research has shown that piglets may absorb immune cells transferred by colostrum independently if they originate from their biological mother or from another sow [29].

### Estimation of MDI transferred to the piglet by dams’ parameters

The most used technique to measure MDA levels in mammary secretions [26, 51–53] and/or antibodies in serum of sows and piglets [54] is the enzyme-linked immunosorbent assay (ELISA). Occasionally, this technique (commercial or in-house methods) has been also used to monitor the presence and quantity of immunoglobulins in other sample types like oral fluids [55, 56]. Alternatively, monitoring CMI is also feasible but mainly restricted to research purposes because the techniques used (for example, enzyme-linked immunosorbent spot [ELISPOT]) are labour-intensive, hard to read and rarely commercialized.

However, the results obtained from measuring antibody levels in the dam are not effective as a tool for estimating MDI transferred later through colostrum and milk [40]. This is mainly because MDI levels in piglets depend on how effective the colostrum and milk intakes are in terms of quantity and timing.

## Early life vaccination of piglets

Different vaccination strategies are used to immunize the populations of swine production (breeding animals, piglets, or both). The vaccination approach will be determined by the pathogenesis and epidemiology of each pathogen. Understanding the infection dynamics and herd immunological status for each infectious agent will be critical to determine the best vaccination strategy.

Early piglet vaccination might imply that piglets still have MDI. Therefore, a potential interference between residual immunity of maternal origin and the vaccine antigen uptake needs to be considered. Piglet MDI may come from natural infection, vaccination or both scenarios (natural infection and vaccination). In the latter case, and especially if sows are vaccinated by late gestation, the amount of measurable MDA transferred can be very high, increasing the risk of interference. Nevertheless, vaccinating sows and piglets in the appropriate timing may yield the best results in terms of herd protection and productivity.

## Effect of MDA on the seroconversion and efficacy of the most used vaccines in the pig industry for pathogens involved in PRDC

Vaccine efficacy interference by MDA is defined as the ability of residual antibodies transferred to piglets by colostrum and/or milk to block or delay the active immunization of the piglet [57]. This interference involves a number of possible mechanisms, including the neutralization of the immunizing antigen [57], the masking of B cell epitopes, and/or the down-regulation of neonatal Ig synthesis by means of the inhibition of B cell maturation and development [15]. Importantly, this concept is mainly considered with intramuscularly (and probably intradermally) applied vaccines that generate systemic and, eventually, mucosal immune responses. However, the degree of potential vaccine efficacy interference due to MDA with vaccines delivered through mucosal surfaces remains unknown [58].

In the presence of high levels of systemic antibody titres at the time of intramuscular vaccination, the most typical interference effect is a reduced or lack of seroconversion [59–61]. However, the key issue would be if this interference in seroconversion translates into lower vaccine effectiveness. The most widely used method of measuring vaccination effectiveness in terms of productive parameters is the calculation of average daily weight gain (ADWG) [62].

Nowadays, piglets are usually vaccinated against several pathogens at early ages. The most common infectious agents involved in PRDC for which piglet vaccines have been developed are detailed in Table 2, including current data on potential interference with vaccine efficacy. Importantly, while interference with vaccine efficacy is often analyzed by comparing experimental vaccinated groups with different antibody titres, it may also be studied by comparing vaccinated groups of various ages.

**Table 2:** Interference of maternally derived antibodies (MDA) with vaccine efficacy in terms of serological and production parameters

Pathogen	Vaccination age (in weeks)	Interference on antibody seroconversion (assay used)^a^	Interference on production parameters (parameters evaluated)^b^	References
SIV	3	Yes (HI assay)	NE	[23]
	Different age groups	Yes (HI assay)	NE	[67]
	16	Yes (HI assay, ELISA)	NE	[68]
PRRSV	3	Yes (ELISA, VNT)	NE	[71]
	3 & 4	Yes (ELISA)	NE	[82]
PCV-2	4	Yes (ELISA)	No (ADWG)	[96]
	3	NE	No (ADWG)	[93]
	3	Yes (IPMA, VNT)	NE	[17]
	3	Yes (ELISA)	NE	[95]
	3	Yes (IPMA)	NE	[97]
	4	Yes (IPMA)	No (ADWG)	[98]
	Different age groups	Yes (ELISA)	Yes (ADWG)	[99]
	4	Yes (ELISA)	NE	[60]
	3	Yes (ELISA)	Only when S/P values at vaccination are extremelly high (ADWG)	[21]
	2	NE	No (ADWG, mortality)	[100]
*M. hyopneumoniae*	1 & 4	Yes (ELISA)	No (ADWG, mortality)	[115]
	2	Yes (ELISA)	NE	[112]
	1 & 4	Yes (NC)	NE	[114]
	1	No (ELISA)	NE	[109]
	1	No (ELISA)	NE	[111]
	1	Yes (ELISA)	NE	[116]
	1	Yes (ELISA)	NE	[113]
*A. pleuropneumoniae*	6	Yes (ELISA)	NE	[128]
	6	Yes (ELISA)	NE	[123]

^a^“Yes” when the age and/or MDA of the animals of study are associated with reduced/retarded active antibody post-vaccinal response; “No” when the age and/or MDA of the animals of study are not attributed to a reduced/retarded active antibody post-vaccinal response; “NE” when serologic parameters are not evaluated. HI assay: Hemagglutination-inhibition assay; VNT: Virus neutralisation test; ELISA: Enzyme-linked immunosorbent assay; IPMA: Immunoperoxidase monlayer assay; NC: Not cited

^b^*NE* Not evaluated; *ADWG* Average daily weight gain

### Swine influenza viruses

Swine influenza viruses (SIV), generally of type A, are the causal agents of swine influenza and a major cause of acute respiratory disease outbreaks in pigs. Nowadays, different subtypes of SIV as H1N1, H1N2, H3N2, and pandemic H1N1 virus are co-circulating worldwide [63]. Their infection can display different clinical forms, ranging from an acute outbreak to an endemic subclinical scenario [64]. Although the epizootic presentation is more aggressive, the endemic one is more common within herds. The virus is spread primarily through direct contact with infectious oronasal secretions [63]. In this scenario, newborn piglets that do not receive MDI are at a high risk of showing clinical signs.

Some studies describing the MDA duration against SIV reported a steadily decline until the age of 10 weeks [23, 65], with an average waning period of 7–8,5 weeks [65]. However, other investigations have reported that MDA in piglets coming from vaccinated sows could persist up to 2–4 months of age under both experimental [66–68] and field [69] conditions.

Currently, the main strategy for controlling SIV infection is vaccination. Nevertheless, in the presence of high MDA levels, previous studies reported interference of early piglet vaccination in terms of reduced post-vaccination humoral response [23, 67] and worse respiratory clinical signs [23, 68]. Interestingly, Kitikoon et al. [23] found that piglet vaccination gave better protection (less fever, lower viral shedding and reduced pneumonia) than MDA against SIV, raising doubts on the common practice of immunizing sows to boost MDI. Information on the putative interference of MDA in vaccine efficacy in terms of production parameters has not been assessed so far.

### Porcine reproductive and respiratory syndrome virus (PRRSV)

Porcine reproductive and respiratory syndrome (PRRS) is a common disease in pigs with one of the major economic impacts in porcine production worldwide [70]. Its etiological agent is PRRSV, a virus that causes reproductive problems in sows and respiratory disorders in pigs of all ages, as well as slower growth and mortality in growing pigs [71]. This virus can also cause a long-lasting infection, as animals can remain contagious even after clinical disease recovery [72, 73]. Since piglets can be infected congenitally or very early in life [74], it is critical to protect them as soon as possible after birth or also to protect against intrauterine infections.

Different studies showed that MDA has a significant protective effect against PRRSV infection in suckling piglets [75, 76], lasting between 2 and 11 weeks [71, 72, 77–82]. In fact, the highest rates of PRRSV detection in sera usually occur between 6 and 8 weeks of age, when MDA have achieved lowest levels in many cases [71, 72, 81]. Therefore, since high levels of MDI may offer a strong protection to the piglets in the first few weeks of life, one of the options is to protect the piglet through dam vaccination [71]. However, piglets gradually lose passive immunity, resulting in a steady supply of PRRSV-susceptible piglets and allowing the virus to spread across pig herds. Under this scenario, piglet vaccination may strengthen the piglet’s own early humoral and cellular immune responses [71].

Since vaccination of sows and piglets is a common strategy for controlling PRRS, it is important to consider the effect the MDI may have on developing an active immune response after vaccination. In this regard, recent investigations have yielded contradictory results. According to Fablet et al. [71] and Renson et al. [82], piglets with high MDA at vaccination had a hindered post-vaccination immunological response for at least 4 weeks. In contrast, Balasch et al. [78] and Jeong et al. [83] showed that vaccines can overcome maternal immunity and piglets as young as one day old can generate a partially protective immune response. It may happen that different vaccines may have different ability to overcome MDI, but side-by-side comparisons in such regard have not been performed.

Noteworthy, up to now, interference of MDI on PRRSV vaccine efficacy in terms of productive parameters has not been studied.

### Porcine circovirus 2 (PCV-2)

Porcine circovirus 2 (PCV-2) is the primary aetiologic agent of porcine circovirus diseases (PCVDs) [84], which include the systemic disease (PCV-2-SD), the reproductive disease (PCV-2-RD), porcine dermatitis and nephropathy syndrome (PDNS) and the subclinical infection (PCV-2-SI) [85–87]. Although PCV-2 is ubiquitous in domestic swine and wild boar, pigs younger than 4 weeks of age are very rarely affected by the disease [88]. This suggest that certain levels of MDI prevent the development of PCV-2-SD in the offspring [89, 90]. However, early infections, including intrauterine infections, do occur in farms in presence or absence of subsequent PCV-2-SD in late nursery or growing pigs [91, 92].

MDA against PCV-2 is known to last between 4 and 12 weeks of age [21, 60, 93–96], and can be fostered by sow vaccination at mid-late gestation. Although a strong maternal immunization is crucial for the newborn piglet protection against infection, when the piglet is vaccinated it may block the vaccine antigen. Indeed, high MDA titres against PCV-2 have been shown to impair an active seroconversion after vaccination [17, 21, 60, 95–99]. However, production parameters such as ADWG did not appear to be jeopardized in similar scenarios [21, 93, 96, 98, 100], suggesting that interference with seroconversion does not always imply a lack of protection. Of note, Feng et al. [21] highlighted that extremely high titres of MDA at vaccination may interfere with production parameters, although it was considered not economically relevant in practical conditions as it is rare to find such high MDA titres in the field. Alternatively, Haake et al. [99] concluded that, regardless the antibody titre at vaccination, immunization at 1 week of age can result in lower production parameters than immunizing later (3 weeks of age). However, in this study, 1 week-old vaccinated piglets had higher antibody values than those vaccinated at 3 weeks of age.

### Mycoplasma hyopneumoniae

*Mycoplasma hyopneumoniae* is the main aetiological agent of enzootic pneumonia [101, 102]. Under experimental conditions, *M. hyopneumoniae* infection can last for up to 240 days [103]. During that time, animals could still shed the pathogen in a slow and silent fashion, which explains the *M. hyopneumoniae* endemic and chronic behaviour on most farms.

The prevalence of *M. hyopneumoniae* infection in piglets at weaning has been, in some studies, correlated with the prevalence and severity of lung lesions in fattening pigs [104, 105]. For this reason, reducing vertical transmission from dams to piglets during lactation period is considered a critical point in *M. hyopneumoniae* control. In consequence, sow vaccination strategies have been proposed as a potential tool to reduce vertical transmission and induce specific antibodies and CMI in sow serum and colostrum. These components would be, in turn, transferred to their suckling piglets and play a role in piglet protection [14, 29, 106–108]. MDA against *M. hyopneumoniae* wane approximately between 2 and 9 weeks [20, 109]. Moreover, piglet vaccination is the most common practice, and it usually takes place around weaning, within the 4 first weeks of life. Nevertheless, some vaccines are licensed to be applied within the first week of life [110] and, consequently, such vaccination takes place in presence of MDI [111].

The influence of MDI on piglet’s vaccination has not been fully elucidated. Whereas some studies reported that the antibody response elicited by vaccination in face of MDI could be reduced or absent [112–115], some others do not describe it [109, 111, 116]. However, lack of seroconversion following vaccination has not been related to worse productive parameters of the piglet so far [115].

### Actinobacillus pleuropneumoniae

*Actinobacillus pleuropneumoniae* is the aetiological agent of porcine contagious pleuropneumonia. Virulent serotypes of this bacteria can cause respiratory distress, anorexia, and fever, with variable degrees of severity depending on the clinical form: peracute, acute, or subacute [117]. Death is frequent in peracute and acute presentations. Subclinical infections can also take place with several *A. pleuropneumoniae* serotypes [118]. This bacterium cannot survive in the environment for long periods of time [119]; therefore, transmission is mostly pig-to-pig via direct contact, both oral and nasal, and, to a lesser extent, via aerosols over short distances [120]. In addition, infected pigs that carry the bacterium silently contribute to the pathogen’s spread [121]. Therefore, a proportion of piglets are exposed to the bacterium early in life, mainly during the suckling period. Subsequently, when MDI wanes by end of the nursery period or during the growing phase, infection is further spread [119].

MDA against *A. pleuropneumoniae*-specific can persist from 2 to 12 weeks of age in the offspring [18, 19, 122–124]. Vaccinating dams against *A. pleuropneumoniae* can be an effective method for improving the herd serological status and, consequently, the amount of acquired colostral antibodies in piglets [125]. Colostral immunity protects piglets against sudden death or peracute outbreaks, but not against infection, as piglet infection with *A. pleuropneumoniae* can occur from 10th day of life even in the presence of MDA [126]. However, high levels of MDI against the pathogen are especially indicated for newborns as they remain seropositive for a longer period and enhances antibody response when they seroconvert due to natural infection [124]. Indeed, Krejci et al. [127] found that the protection of the piglet could be even better if, in addition to having specific colostrum-derived antibodies, it was pre-infected with low infection doses.

Vaccine manufacturers recommend vaccination in late nursery and/or early fattening pigs for protection against *A. pleuropneumoniae* [123]. However, vaccination at those ages is a concern due to interference of MDA that may still be present. Tumamao et al. [128] found that vaccinating piglets against *A. plueropneumoniae* in the presence of low levels of MDA induces a significant antibody response, but no comparison with animals with high MDA levels was made in the same study. On the other hand, Jirawattanapong et al. [123] found no antibody response after vaccination of pigs in presence of high MDA titres at 6 and 10 weeks of age. Therefore, it seems that vaccination against *A. pleuropneumoniae* should not be used during the first weeks of life to prevent an impairment of post-vaccinal antibody response [119].

Notably, evident interference of MDI on *A. pleuropneumoniae* vaccine effectiveness in terms of productive parameters has not been found in published studies. However, all marketed products against this pathogen indicate vaccination from 6 weeks of age onwards, which suggests vaccine efficacy interference if administered to earlier ages still with MDI (European Medicines Agency—https://ema.europa.eu).

## Discussion

Vaccination can be applied to sows, piglets or both populations. Sow immunization prior to farrowing enhances MDI transferred to offspring through colostrum and milk. Piglet vaccination at early ages is directed to immunize them before they become infected naturally. The optimal moment would be when MDA levels are high enough to protect the piglet, but low enough to minimize the interference with vaccine antigen. That is why the third alternative, vaccinating both collectives, is the one at a higher risk of interference with vaccine efficacy in the piglet.

Several studies have assessed the amount and duration of MDA to properly ascertain the best timing to apply the vaccine to the piglet. However, the exact duration of MDA for each pig or groups of pigs is virtually impossible to be assessed under field conditions, since it is dependent on the sow’s serological status at farrowing and on the piglet colostrum intake. Therefore, it is expectable to have high individual variability amongst sows and even within piglets from the same litter.

The most likely situation is that piglets are vaccinated in the presence of an unknown level of MDA. This scenario would imply a potential risk of interference between these antibodies and the vaccine antigen intake. In this regard, it is worth noting that interference might be assessed from two different perspectives: interference on seroconversion and interference on vaccine efficacy in terms of production parameters (mainly ADWG). Several studies on PRDC pathogens reviewed in this work have shown that MDA may interfere with seroconversion, particularly when the titres of systemic antibodies were high at the time of piglet vaccination. Therefore, it might be interesting in some cases to wait for MDA declining and vaccinate piglets beyond 3–4 weeks of age, or even later. In contrast, when vaccine efficacy was also evaluated, the interference was shown in terms of seroconversion elicited by the immunization, but it was rarely translated in worse productive parameters. However, it must be considered that lack of demonstrated commercial vaccine effectiveness is unlikely to be published in the literature. Furthermore, it would be also worthy to investigate if the existence of antigen-specific CMI of maternal origin in the neonatal piglet can influence the development of vaccine-induced immunity, particularly in those cases where the piglet is vaccinated very early [129].

All in all, based on the existing literature, early piglet vaccination could be considered as an option to protect the piglet in terms of reduction of clinical signs and improving performance parameters, notwithstanding the potential serological interference issue.

## Conclusion

According to the literature, MDA transferred from sows to offspring could potentially interfere with the development of an active humoral immune response when vaccines are applied at the recommended age for most of the PRDC pathogens. However, no conclusive interference has been shown regarding performance parameters based on the existing published literature.

## Data Availability

All data used in this review work has been obtained from public scientific databases, available elsewhere.
